# Supplementary material for: Reward signaling in a recurrent circuit of dopaminergic neurons and peptidergic Kenyon cells
Source: Nat Commun. 2019 Jul 15;10:3097. doi: 10.1038/s41467-019-11092-1 (PMC6629635; doi:10.1038/s41467-019-11092-1)
Supplement: Supplementary file 1 — Supplementary Information [file 41467_2019_11092_MOESM1_ESM.pdf]

Supplementary Information

Reward signaling in a recurrent circuit of dopaminergic neurons and peptidergic Kenyon cells

Lyutova et al.

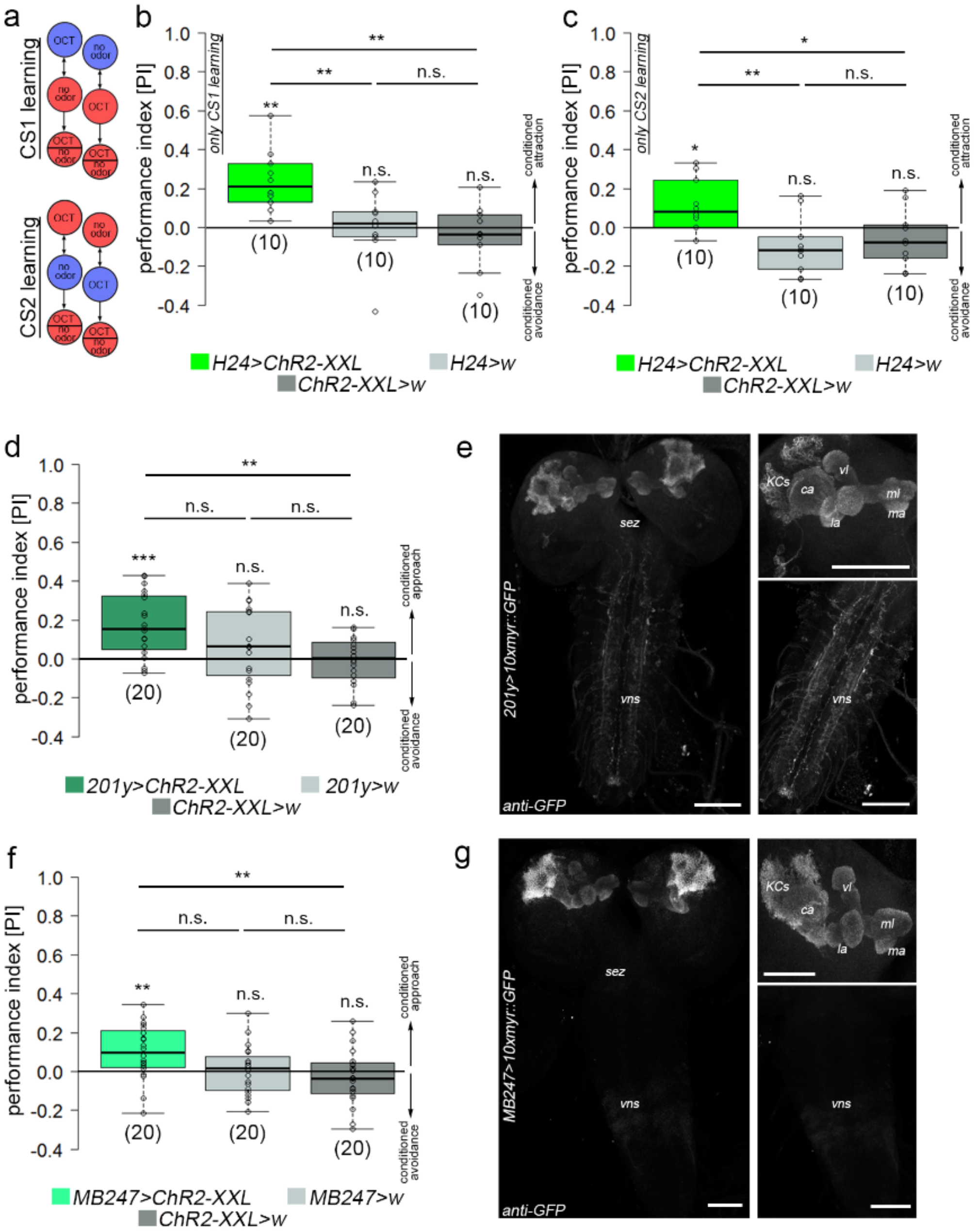

Supplementary Figure 1 (Panels a-g): Optogenetic activation of KCs induces memory formation

(a-c) Performance scores were independent of whether the first odor (CS1+; OCT or “no odor”) or the second odor (CS2+; OCT or “no odor”) was coupled to optogenetic activation of KCs during KC-substitution learning (*H24>ChR2-XXL*). (d) Optogenetic activation of KCs using *201y-Gal4* induces appetitive memory expression. (e) *201y-Gal4* labels around 315 KCs and only few cells in the VNS. (f) Optogenetic activation of KCs using *MB247-Gal4* induces appetitive memory expression. (g) *MB247-Gal4* is expressed in around 341 KCs. *10xmyr::GFP*: 10xUAS-*myristoylated green-fluorescent protein;* ca: calyx; CS: conditioned stimulus; KCs: Kenyon cells; la: lateral appendix; ma: medial appendix; ml: medial lobe: OCT: octanol; sez: subesophageal

zone; vl: vertical lobe: vns: ventral nervous system. The number below the boxplots refers to the N number. A pairwise Student´s t test or pairwise Wilcoxon test (both including Bonferroni-Holm correction) was used. Significance levels: p>0.05 n.s., p<0.05 *, p<0.01 **, p<0.001 ***. Data is presented as box plots, with 50% of the values of a given genotype being located within the box, and whiskers represent the entire set of data. No data was excluded. Outliers are indicated as open circles. The median performance index is indicated as a thick line within the box plot.

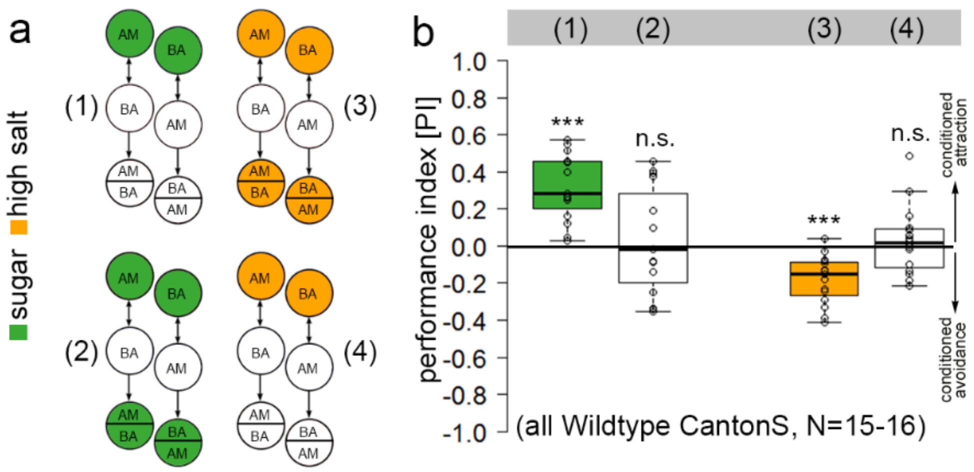

Supplementary Figure 2 (Panels a-b): Memory expression is context-dependent in larvae

(a-b) Wildtype larvae express appetitive olfactory memories only in absence of the appetitive US (e.g. sugar (green)) in the test situation, while aversive memory expression is only present when the aversive gustatory US (e.g. high salt (orange)) is present in the test situation. AM: amylacetate; BA: benzyaldehyde. A pairwise Student´s t test or pairwise Wilcoxon test (both including Bonferroni-Holm correction) was used. Significance levels: p>0.05 n.s., p<0.05 *, p<0.01 **, p<0.001 ***. Data is presented as box plots, with 50% of the values of a given genotype being located within the box, and whiskers represent the entire set of data. No data was excluded. Outliers are indicated as open circles. The median performance index is indicated as a thick line within the box plot.

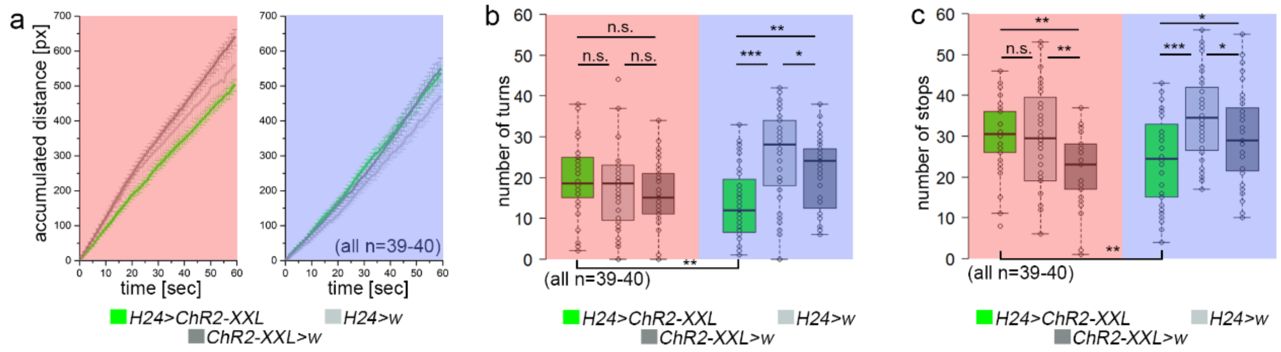

Supplementary Figure 3 (Panels a-c): Optogenetic activation of KCs affects larval locomotion.

(a) General locomotion was unaffected by optogenetic activation of all KCs as *H24>ChR2-XXL* larvae crawled normal distances over time compared to genetic controls. Experimental larvae showed a reduced number of turns (b) and stops (c) due to blue light exposure. A pairwise Student´s t test or pairwise Wilcoxon test (both including Bonferroni-Holm correction) was used. Significance levels: p>0.05 n.s., p<0.05 *, p<0.01 **, p<0.001 ***. Data is presented as box plots, with 50% of the values of a given genotype being located within the box, and whiskers represent the entire set of data. No data was excluded. Outliers are indicated as open circles. The median performance index is indicated as a thick line within the box plot.

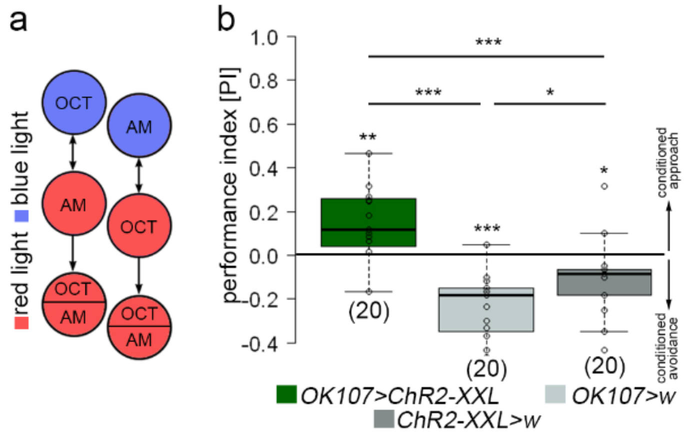

Supplementary Figure 4 (Panels a-b): KC-substitution learning in the two-odor reciprocal training regime.

Conditional optogenetic activation of KCs is sufficient to induce an appetitive memory in a two-odor reciprocal training regime (a) using *OK107-Gal4* (b). AM: amylacetate; OCT: octanol. The number below the boxplots refers to the N number. A pairwise Student´s t test or pairwise Wilcoxon test (both including Bonferroni-Holm correction) was used. Significance levels: p>0.05 n.s., p<0.05 *, p<0.01 **, p<0.001 ***. Data is presented as box plots, with 50% of the values of a given genotype being located within the box, and whiskers represent the entire set of data. No data was excluded.

Outliers are indicated as open circles. The median performance index is indicated as a thick line within the box plot.

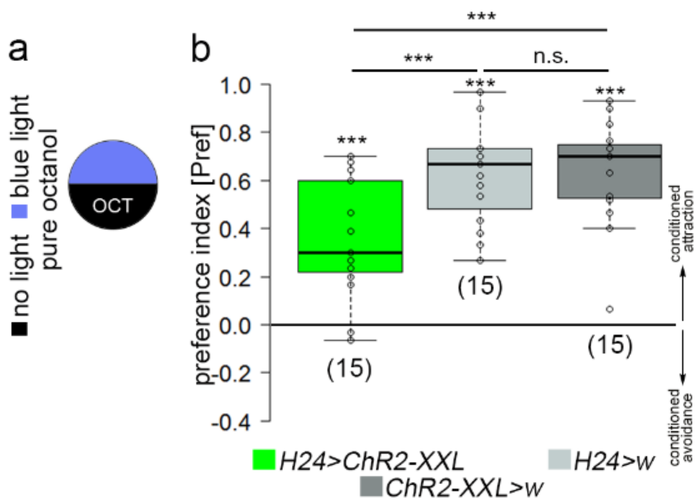

Supplementary Figure 5 (Panels a-b): Optogenetic activation of KCs induces internal reward signaling.

H24>ChR2-XXL larvae show a reduced light avoidance compared to genetic controls when they are challenged to decide between their innate OCT and darkness preference versus an optogenetically induced internal reward signaling (b). OCT: octanol. The number below the boxplots refers to the N number. A pairwise Student´s t test or pairwise Wilcoxon test (both including Bonferroni-Holm correction) was used. Significance levels: p>0.05 n.s., p<0.05 *, p<0.01 **, p<0.001 ***. Data is presented as box plots, with 50% of the values of a given genotype being located within the box, and whiskers represent the entire set of data. No data was excluded. Outliers are indicated as open circles. The median performance index is indicated as a thick line within the box plot.

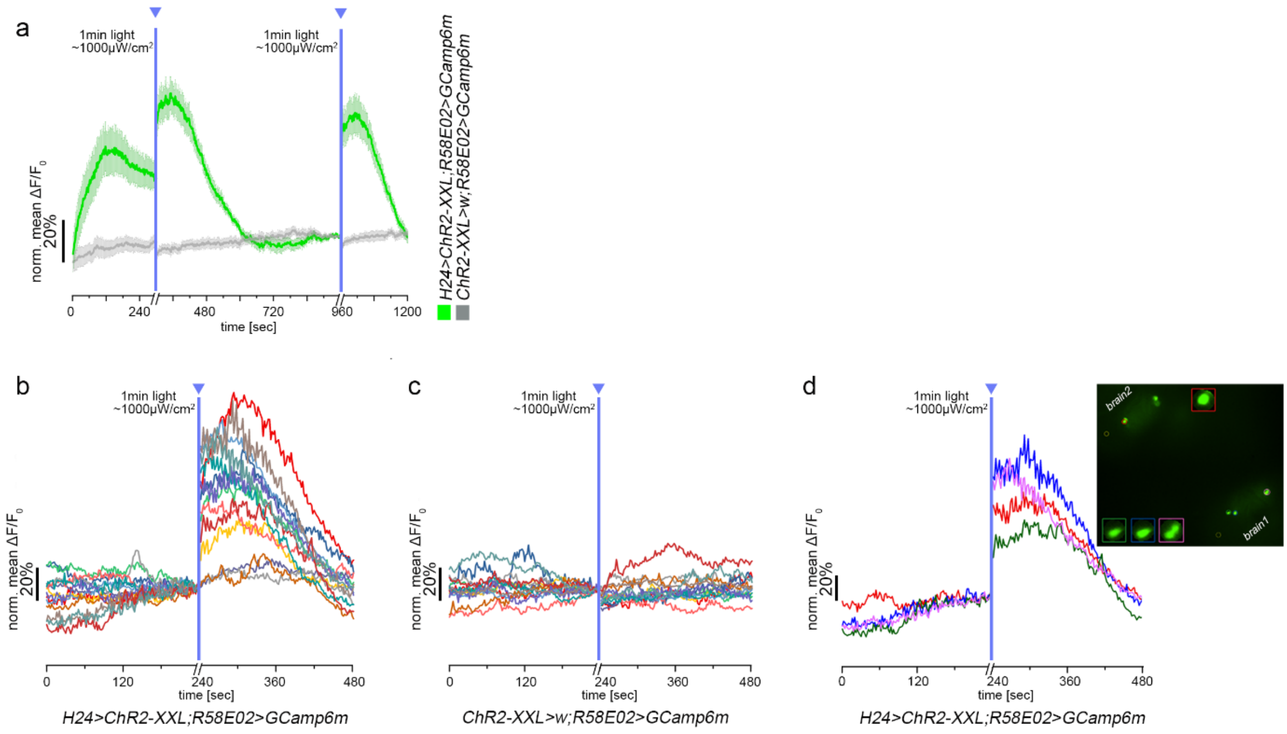

Supplementary Figure 6 (Panels a-d): Optogenetically-induced memory expression is based on KC-to-pPAM signaling.

(a) Fluorescence activity monitored in pPAM neurons in response to optogenetic activation of KCs (*H24>ChR2-XXL;R58E02>GCamp6m, green*). In contrast to the control group (grey) fluorescence activity increases immediately to light (475nm; ~500 µW cm^{-2}). However, exposure to a one minute continuous light pulse (475nm; ~1000 µW cm^{-2}) strongly increased fluorescence activity in pPAM neurons (at 300s and at 960s). Importantly, fluorescence activity in pPAM neurons returned to baseline when specimen are exposed to ~500 µW cm^{-2} in between light pulses. (b-c) 14 single traces of fluorescence activity in pPAM neurons in *H24>ChR2;R58E02>GCamp6m* (b) and in controls (c). Data 240s before and 240s after the second light pulse are plotted. (d) Expression of GCamp6m in pPAM neurons. Example traces (240s before and after the second light pulse) and corresponding drawing of ROIs around pPAM cell bodies. Error bars (a) represent the standard error of the mean. ROI: Region of interest.

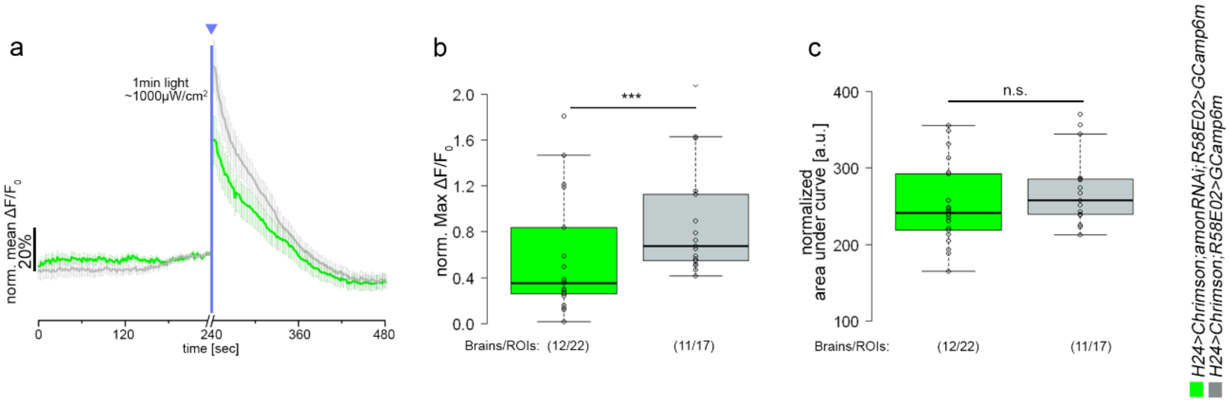

Supplementary Figure 7 (Panels a-c): Optogenetically-induced memory expression is based on peptidergic signaling.

(a-c) Fluorescence activity in pPAM neurons in response to optogenetic activation of MB KCs using *UAS-Chrimson* (illumination with blue light (475nm)) is significantly reduced in specimen expressing *amontillado* RNAi in KCs (green) compared to genetic controls (grey). (b) Significant reduction of maximal values in pPAM cells after activation of *amontillado* RNAi expressing KCs. (c) No significant differences in the area under the curve. A pairwise Student´s t test or pairwise Wilcoxon test (both including Bonferroni-Holm correction) was used. Significance levels: p>0.05 n.s., p<0.05 *, p<0.01 **, p<0.001 ***. Error bars (a) represent the standard error of the mean. Data is mainly presented as box plots, with 50% of the values of a given genotype being located within the box, and whiskers represent the entire set of data. No data was excluded. Outliers are indicated as open circles. The median performance index is indicated as a thick line within the box plot.
