# Supplementary material for: Reward signaling in a recurrent circuit of dopaminergic neurons and peptidergic Kenyon cells
Source: Nat Commun. 2019 Jul 15;10:3097. doi: 10.1038/s41467-019-11092-1 (PMC6629635; doi:10.1038/s41467-019-11092-1)
Supplement: Supplementary file 3 — Reporting Summary [file 41467_2019_11092_MOESM3_ESM.pdf]

nature research

Corresponding author(s): Dennis Pauls

Last updated by author(s): 17.06.2019

Reporting Summary

Nature Research wishes to improve the reproducibility of the work that we publish. This form provides structure for consistency and transparency in reporting. For further information on Nature Research policies, see Authors & Referees and the Editorial Policy Checklist.

Statistics

For all statistical analyses, confirm that the following items are present in the figure legend, table legend, main text, or Methods section.

n/a	Confirmed	
☐	☒	The exact sample size (n) for each experimental group/condition, given as a discrete number and unit of measurement
☐	☒	A statement on whether measurements were taken from distinct samples or whether the same sample was measured repeatedly
☐	☒	The statistical test(s) used AND whether they are one- or two-sided<br>*Only common tests should be described solely by name; describe more complex techniques in the Methods section.*
☒	☐	A description of all covariates tested
☐	☒	A description of any assumptions or corrections, such as tests of normality and adjustment for multiple comparisons
☐	☒	A full description of the statistical parameters including central tendency (e.g. means) or other basic estimates (e.g. regression coefficient) AND variation (e.g. standard deviation) or associated estimates of uncertainty (e.g. confidence intervals)
☒	☐	For null hypothesis testing, the test statistic (e.g. F, t, r) with confidence intervals, effect sizes, degrees of freedom and P value noted<br>*Give P values as exact values whenever suitable.*
☒	☐	For Bayesian analysis, information on the choice of priors and Markov chain Monte Carlo settings
☒	☐	For hierarchical and complex designs, identification of the appropriate level for tests and full reporting of outcomes
☒	☐	Estimates of effect sizes (e.g. Cohen's d, Pearson's r), indicating how they were calculated

Our web collection on statistics for biologists contains articles on many of the points above.

Software and code

Policy information about availability of computer code

Data collection	n/a
Data analysis	No specific software or code was used in this study.

For manuscripts utilizing custom algorithms or software that are central to the research but not yet described in published literature, software must be made available to editors/reviewers. We strongly encourage code deposition in a community repository (e.g. GitHub). See the Nature Research guidelines for submitting code & software for further information.

Data

Policy information about availability of data

All manuscripts must include a data availability statement. This statement should provide the following information, where applicable:

- Accession codes, unique identifiers, or web links for publicly available datasets
- A list of figures that have associated raw data
- A description of any restrictions on data availability

All data is available from the authors on request. Connectomic analyses are based on data available with Eichler et al. (reference 11)

Field-specific reporting

Please select the one below that is the best fit for your research. If you are not sure, read the appropriate sections before making your selection.

☒ Life sciences ☐ Behavioural & social sciences ☐ Ecological, evolutionary & environmental sciences

For a reference copy of the document with all sections, see nature.com/documents/nr-reporting-summary-flat.pdf

Life sciences study design

All studies must disclose on these points even when the disclosure is negative.

Sample size	Sample sizes were based on previous reports on chemosensory learning and memory with moderate to weak effect sizes (Pauls et al 2010, Pauls et al 2015, Rohwedder et al 2015) (references 3,30,52)
Data exclusions	No data was excluded.
Replication	In case replicates were done, findings could be reproduced (e.g. Fig 1+4+6).
Randomization	not relevant for this study.
Blinding	Experimentators were -at least- partially (~ 1/4 of the experiment) blind to genotypes.

Reporting for specific materials, systems and methods

We require information from authors about some types of materials, experimental systems and methods used in many studies. Here, indicate whether each material, system or method listed is relevant to your study. If you are not sure if a list item applies to your research, read the appropriate section before selecting a response.

Materials & experimental systems

n/a	Involved in the study
☐	☒ Antibodies
☒	☐ Eukaryotic cell lines
☒	☐ Palaeontology
☐	☒ Animals and other organisms
☒	☐ Human research participants
☒	☐ Clinical data

Methods

n/a	Involved in the study
☒	☐ ChIP-seq
☒	☐ Flow cytometry
☒	☐ MRI-based neuroimaging

Antibodies

Antibodies used	(1) polyclonal rabbit anti-GFP antibody (A6455, Molecular Probes, dilution 1:1000); (2) monoclonal mouse anti-GFP (A11120, Molecular Probes, dilution 1:250); (3) polyclonal rabbit anti-sNPFp (Johard et al., 2008, Nässel et al., 2008, dilution 1:1000); (4) secondary antibody solution containing goat anti-rabbit Alexa 488 (Molecular Probes, dilution 1:250); (5) goat anti-mouse DyLight 488 (Jackson ImmunoResearch, dilution 1:250); (6) goat anti-rabbit Alexa 635 (Molecular Probes, dilution 1:250)
Validation	(1) The antibody was succesfully used in Drosophila and validated in Busch, Selcho, Ito, Tanimoto 2009, The Journal of Comparative Neurology; (2) The antibody was succesfully used in Drosophila and validated in Rohwedder, Selcho, Chassot, Thum 2015, The Journal of Comparative Neurology;<br>(3) The antibody was succesfully used in Drosophila and validated in i.a. Johard, Enell, Gustafsson, Trifilieff, Veenstra, Nässel 2008, The Journal of Comparative Neurology;

Animals and other organisms

Policy information about studies involving animals; ARRIVE guidelines recommended for reporting animal research

Laboratory animals	Drosophila melanogaster; Driver lines used in this study were H24-Gal4 (chromosome III), OK107-Gal4 (IV), 201y-Gal4 (II), MB247-Gal4 (III), R58E02-Gal4 (III), and R58E02-LexA (II). UAS lines included in this study were 10xUAS-IVS-myr::GFP (II), UAS-mCD8::GFP (II), UAS-ChR2-XXL (II), UAS-AChR1-RNAi (III),UAS-AChR1-RNAi (III), UAS-AChR5-RNAi (III), UAS-AChR6-RNAi (III), UAS-amon-RNAi78b (III), UAS-amon-RNAi28b (II), UAS-sNPFR-RNAi (III), 20xUAS-CsChrimson (I), UAS-DopR1-RNAi (III), LexAop-reaper (III), and 13xLexAop-IVS-GCamp6m,20xUAS-CsChrimson (III). Genetic controls were obtained by crossing Gal4-driver/Lex-driver and UAS-effector/LexAop-effector lines to w1118 (see Methods Section)
Wild animals	n/a
Field-collected samples	n/a
Ethics oversight	n/a

Note that full information on the approval of the study protocol must also be provided in the manuscript.
